# Intrinsic interaction inferred oxidative stress and apoptosis by Biosurfactant-microplastic hybrid reduces coordinated *in vivo* biotoxicity in zebrafish (*Danio rerio*)

**DOI:** 10.1016/j.mtbio.2025.101466

**Published:** 2025-01-07

**Authors:** Utsa Saha, Aishee Ghosh, Adrija Sinha, Aditya Nandi, Sudakshya S. Lenka, Abha Gupta, Shalini Kumari, Anu Yadav, Mrutyunjay Suar, Nagendra Kumar Kaushik, Vishakha Raina, Suresh K. Verma

**Affiliations:** a School of Biotechnology, KIIT University, Bhubaneswar, 751024, India; b Department of Physics and Astronomy, Uppsala University, Box 516, Uppsala SE-751 20, Sweden; c Markham College of Commerce, Vinoba Bhave University, Hazaribagh, Jharkhand 825001, India; d Plasma Bioscience Research Center, Department of Electrical and Biological Physics, Kwangwoon University, 01897 Seoul, South Korea

**Keywords:** Microplastics, Biosurfactant, Zebrafish, Eco-toxicity, Oxidative stress, Apoptosis

## Abstract

The proliferation of microplastics (μP) in aquatic environments poses a significant threat to ecosystem health, with repercussions extending to aquatic organisms and potentially to human health. In this study, we investigated the efficacy of a novel biosurfactant-microplastic (BSμP) hybrid in reducing *in vivo* green bio-toxicity of microplastics (μP) induced by oxidative stress and apoptosis in zebrafish (*Danio rerio*). Microplastics, ubiquitous in aquatic environments, were hybridised with Biosurfactant to evaluate their potential mitigating effects. A stable BSμP was formed with zeta potential of −10.3 ± 1.5 mV. Exposure of zebrafish embryos to μP resulted in increased oxidative stress markers, including elevated levels of reactive oxygen species and induced apoptosis, as evidenced by increased expression of apoptotic markers and morphological changes in embryonic zebrafish. However, the BSμP hybrid significantly ameliorated the observed toxic effects with reduced levels of oxidative stress markers and apoptotic activity. This effect was deduced as the intrinsic effects of hybridisation, which likely mitigated the bioavailability and toxicity of μP by reducing their molecular interaction with metabolic proteins like Sod1 and p53 through less accumulation and internalisation. Overall, our findings highlight the potential of BSμP as a promising approach for mitigating the ecological impacts of microplastic pollution.

## Highlights

• Biosurfactant (BS) can hybridize with Micro-polystyrene (μP) to reduce the concern of environmental toxicity.
• BS and μP can hybridize in natural condition to form BSμP hybrid.
• BSμP induces lower deformities, oxidative stress and apoptosis than BS and μP in zebrafish embryos.
• Bioaccumulation and Intrinsic atomic interaction of BSμP with Zhe1a causes physiological abnormalities.
• Proximal discrepancies in intrinsic atomic interaction of BS, μP and BSμP determines in vivo biotoxicity.

## Graphical abstract

**Figure:**
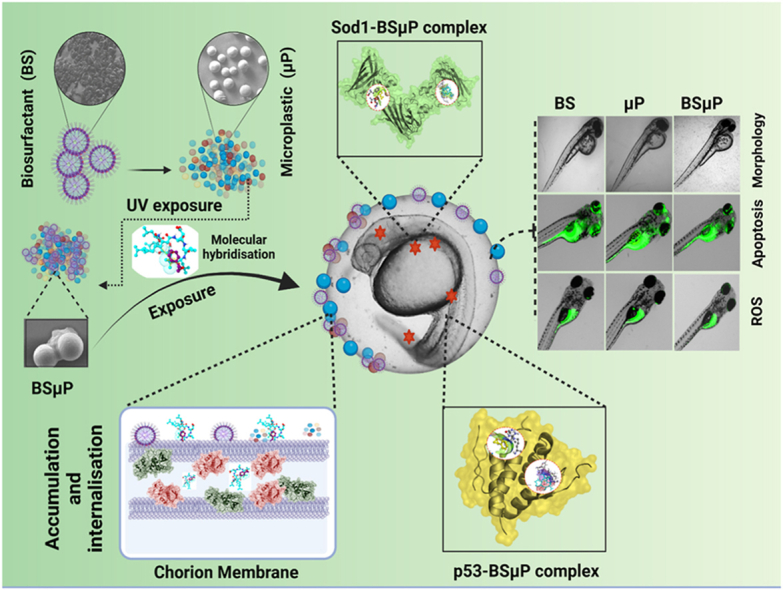

## 1 Introduction

Plastics, widely used since the 1950s, pose serious environmental threats despite their convenience and versatility. Microplastics (μPs), tiny particles (1–5000 μm) formed from larger plastics, have become a global challenge, contaminating terrestrial and aquatic ecosystems [[1], [2], [3], [4], [5], [6]] Coined by marine ecologist Richard Thompson, “microplastic" refers to particles originating from sources like wastewater, cosmetics, and industrial activities. μPs exist in five forms: microbeads, fibers, fragments, nurdles, and foam, categorized as primary (designed for industrial use) or secondary (formed by the breakdown of larger plastics) [[7], [8], [9], [10], [11]]. Natural processes like UV exposure and ocean waves degrade plastics into brittle fragments over time, but these μPs persist for years, causing ecological harm. Scientists are exploring solutions, including bioremediation, which uses microorganisms with enzymatic capabilities to degrade plastic polymers [12]. This approach shows promise in mitigating microplastic pollution, offering a potential pathway to reduce their environmental impact and protect ecosystems [13].

Surfactants, as the term implies, are substances that when added to a liquid, reduce its surface tension, thereby increasing its spreading and wetting properties. Biosurfactants (BSs) are surface-active compounds produced extracellularly in the environment by microorganisms like bacteria, filamentous fungi, or yeast, especially when they are required to grow on water-immiscible substrates. Microorganisms synthesize biosurfactants to facilitate their growth on water-immiscible substrates, as well as to aid in the mobilization and uptake of nutrients from their surroundings. These amphiphilic molecules possess a unique molecular structure comprising hydrophilic and hydrophobic moieties, which confer upon them the ability to reduce surface tension and enhance the solubilization and dispersion of hydrophobic substances in aqueous environments. Unlike their synthetic counterparts, biosurfactants are biodegradable, environmentally friendly, and often exhibit superior performance under extreme conditions, making them attractive candidates for various industrial, agricultural, and environmental applications. One of the most intriguing aspects of biosurfactants is their versatility and multifunctionality. These compounds exhibit a wide range of surface-active properties, including emulsification, foaming, wetting, and dispersing, which find applications across numerous industries such as oil and gas, agriculture, food processing, pharmaceuticals, and bioremediation [[14], [15], [16], [17]]. Furthermore, biosurfactants have shown great promise in environmental remediation efforts. These molecules, produced by microbes exhibit surfactant properties that enhance the bioavailability and biodegradation of organic pollutants [18].

Current environmental risk assessments predominantly concentrate on single substances rather than the complex mixtures formed by the interaction of multiple compounds [[19], [20], [21]]. Although various studies have investigated the individual effects of microplastics and biosurfactants on aquatic organisms, there is a notable gap in research regarding their combined toxicological impact under coexistence scenarios. To our knowledge, no comprehensive study has explored the synergistic or antagonistic effects of microplastics and biosurfactants on aquatic ecosystems. Given the widespread occurrence of both microplastics and biosurfactants in aquatic environments, elucidating their interactions and understanding their ecological consequences is crucial for developing effective environmental management and mitigation strategies [22]. This necessitates further investigation into the combined toxicological effects of microplastics and biosurfactants to safeguard aquatic ecosystems and ensure sustainable environmental stewardship.

Zebrafish have been recognized as one of the popular invivo models for toxicological studies. Zebrafish and their embryos are valuable in toxicological studies due to their rapid development, optical transparency, genetic similarity to humans, and high-throughput capabilities. They enable cost-effective, non-invasive observation of developmental processes and toxic effects, making them ideal for environmental and pharmaceutical testing [23]. In this study, we aimed to investigate the formation of microplastic-biosurfactant (BSμP) hybrids and evaluate their toxicological effects on zebrafish embryos. Commercial μP beads were mixed with BS extracted from the bacterium *Brevibacterium casei* (strain LS14) in various ratios to form a hybrid by UV crosslinker. Through a combination of experimental approaches including UV–Visible spectrophotometry, dynamic light scattering (DLS), and scanning electron microscopy (SEM), the characterization of the formation of BSμP hybrid was done. Zebrafish embryos were subjected to varying concentrations of BSμP treatment, and their developmental and morphological parameters were assessed. Additionally, cellular analyses focusing on reactive oxygen species (ROS) and apoptosis were conducted using flow cytometry (FACS) to elucidate the toxicological impacts of BSμP exposure. The results obtained indicated an increase in ROS and apoptosis with increasing concentration of BSμP treatment, depicting negative impacts on zebrafish's embryonic development compared to only BS and μP treatment. These results underscore the importance of elucidating the combined toxicological effects of microplastics and biosurfactants on aquatic organisms and the need for further research to inform effective pollution control measures and environmental management strategies. By expanding our understanding of the interactions between microplastics and biosurfactants, we can develop targeted interventions to mitigate microplastic pollution and safeguard aquatic ecosystems for future generations.

## 2 Materials and methods

### 2.1 Microplastic and microbial surfactant

Commercial microplastic (μP) beads (BD FACSTM 7-color setup beads) having polystyrene nature produced by BD Biosciences, India, were employed in the study. These beads were stored within a temperature range of 20 °C–80 °C. To prepare the stock solution, two microplastic beads were suspended in 1 ml of distilled water and vigorously vortexed until a uniform mixture was achieved. The biosurfactant (BS) obtained from the bacterium *Brevibacterium casei*, known for its aerobic gram-positive rod-shaped morphology, was provided by Dr. Khusboo Kumari for the purpose of forming hybrids with microplastics (μP) [24]. Extraction, purification, and characterization of the microbial surfactant were carried out, with nuclear magnetic resonance (NMR) analysis revealing the presence of chemical functionalities including amide, carboxy, and methoxy groups [24]. The biosurfactant was dissolved in methanol and stored at a temperature of 4 °C.

### 2.2 Preparation of microplastic – biosurfactant (BSμP) hybrid

For the synthesis of the BSμP hybrid, separate working solutions were prepared containing 5 mg/ml of the biosurfactant (BS) and 1000 μl of microplastic (μP) solution (comprising 2 μP beads suspended in 1 mL of distilled water). These solutions were then mixed in various ratios of 1:2, 1:3, 1:4, and 1:5. Following thorough mixing by vortexing, UV treatment was administered to all these different ratios using a UV crosslinker (UVP laboratories) for 1 h to facilitate hybrid formation. To identify the optimal ratio for hybrid formation, all four hybrid samples were subjected to centrifugation at 10,000 rpm for 10 min. After removing the supernatant, the weight of the pellets was measured to determine the highest concentration of the formed hybrid (with the 1:2 ratio yielding the maximum amount of hybrid). This ratio was selected for further experimentation to generate different concentrations ranging from 5 to 500 μg/mL for subsequent treatment samples in zebrafish embryos.

### 2.3 Physicochemical characterization of MP-BS hybrid

Utilizing various physical techniques including UV–Visible spectroscopy, Dynamic light scattering (DLS), and Scanning electron microscopy (SEM), the physicochemical properties of the synthesized BSμP hybrids, and microplastics (μPs) and biosurfactants (BSs), were investigated. The optical characteristics of BSμP hybrids were assessed by analysing the SPR peak obtained from UV–Visible spectral scans within the range of 200 nm–800 nm using a UV–Visible spectrophotometer (Cary 60, Agilent Technologies, USA). The hydrodynamic diameter and zeta potential of μP, BS, and BSμP hybrids in an aqueous medium were determined using the Zetasizer (Malvern, UK) to evaluate their size and stability, respectively. SEM imaging (Zeiss) was employed to measure and validate their size and intricate morphology after all samples were converted to their dried forms.

### 2.4 Maintenance of zebrafish and breeding

The research adhered to the relevant animal care guidelines and regulations stipulated by the Institutional Animal Ethics Committee (IAEC) at KIIT University. Zebrafish specimens were procured from a local aquarium fish vendor in Bhubaneswar and housed in an aquarium setting (Aquaneering). The setup was acclimatized for 24 h by using fish water prepared by mixing 75 g of NaHCO_3_, 18 g of sea salt, and 8.4 g of CaSO_4_ per 1000 ml of water. Breeding conditions were optimized by maintaining a male-to-female ratio of 1:2 and regulating photoperiodism with 14 h of light and 10 h of darkness. The water temperature was maintained at a constant 26 ± 2 °C. The zebrafishes were provided with a diet enriched with bloodworms to stimulate breeding activity. Eggs were collected early in the morning, thoroughly rinsed with system water, and transferred to egg water (0.6 g of sea salt in 1 L of distilled water supplemented with 100 μl of methylene blue). This egg water was utilized to ensure the proper maintenance and growth of the zebrafish embryos.

### 2.5 *In vivo* biocompatibility analysis

All experiments concerning the biocompatibility of the BSμP hybrid on zebrafish embryos were conducted following the Institutional Animal Ethics Committee (IAEC) guidelines and the applicable laws of KIIT University. *In vivo* cytotoxicity assessments of the hybrid, as well as commercial microplastics (μPs) and biosurfactants (BSs), on zebrafish embryos were carried out following the protocol outlined by Ref. [25]. The assays utilized commercially purchased μP beads and biosurfactants obtained from microbial sources, aiming to compare and evaluate the biocompatibility of the synthesized BSμP hybrid. 24 zebrafish embryos at a developmental stage of 24 h post-fertilization (hpf) were treated with μPs, BSs, and BSμP hybrids in egg water at concentrations ranging from 5 μg/ml to 500 μg/ml. The concentration of the μPs, BSs, and BSμP hybrids for treatment was chosen given the reported toxicity of BS and μPs in the previously reported literature. The experimental setup was maintained at a temperature of 28 ± 1 °C. Microscopic observations were conducted every 24-h interval to assess morphological and developmental changes in zebrafish embryos resulting from the applied treatments. The hatching rate, calculated as the proportion of embryos that had hatched at each interval compared to the control group, and the mortality rate of the treated embryos, reported as the proportion of deceased embryos at each interval relative to the untreated group, were recorded. Each experiment was replicated three times to ensure precision. Utilizing an inverted bright-field microscope (Zen Zeiss), images of the treated and untreated zebrafish embryos at various early developmental stages were captured, and morphological changes were analyzed.

### 2.6 Oxidative stress analysis by H_2_DCFDA staining

To assess the induction of oxidative stress in zebrafish embryos, the production of reactive oxygen species (ROS) in cells resulting from exposure to microplastics (μP), biosurfactants (BS), and the synthesized BSμP hybrid was investigated independently [26]. This was accomplished by treating zebrafish embryos with commercial μP, BS, and the BSμP hybrid separately for a period of 72 h, after which the embryos were sacrificed by exposure to −20 °C. Subsequently, the embryos were sonicated to generate a single-cell suspension. Following a 30-min dark incubation with 1.25 mg/l of the ROS indicator H2DCFDA fluorescent dye, the stained cell suspension was analyzed using a flow cytometer (Acoustic flow cytometer, Applied Biosystems). The resulting data were visualized in a histogram format. To gate out the debris that was present in the suspension, an FSC and SSC dot plot was utilized in the flow cytometer.

### 2.7 Acridine orange staining for apoptosis analysis

Using acridine orange (AO) staining, apoptosis analysis was conducted on zebrafish embryos [27]. After being treated for 72 h with μP, BS, and BSμP both untreated and treated zebrafish embryos were sacrificed by exposure to −20 °C. Subsequently, the embryos were sonicated to generate a single-cell suspension. Then they underwent a 20-min exposure to 5 μg/ml AO and were subsequently kept in darkness for 30 min. To evaluate apoptosis levels in zebrafish embryos resulting from exposure to microplastics (μP), biosurfactant (BS), and BSμP hybrid at various concentrations, the stained cell suspension was examined using a flow cytometer (Acoustic flow cytometer, Applied Biosystems). The resulting data was presented in the form of a histogram.

### 2.8 Real-time PCR analysis

The estimation of gene expression in embryos exposed to BS, μP, and BSμP was done using real-time PCR analysis to understand the alteration in expression of SOD1/sod1 and TP53/tp53 proteins in Embryonic zebrafish [28]. The samples for analysis were collected from the cell-suspension of unexposed and exposed embryos with 25, 50, 100, and 250 μg/mL of BS, μP, and BSμP, obtained by sonication. The RNA isolation of RNA was performed using Trizol reagent (Ambion, Foster City, CA) according to the manufacturer's instructions. RNase-free DNase I (Fermentas) treatment was executed to the samples, and cDNA synthesis was done using the Hi-cDNA Synthesis Kit (HIMEDIA, Mumbai, India). The quantity and purity of RNA was estimated by Nanodrop (Colibri Microvolume Spectrometer). For each sample, qPCR analysis was carried out by the KAPA SYBR FAST qPCR Master Mix (2X) (KAPA BIOSYSTEMS, Wilmington, MA) with proper cDNA dilution as a template. The presence of any genomic DNA was checked using DNase-treated RNAse, run as a control. The normalization of the genes' expression (SOD1/sod1 and TP53/tp53) was done using 18S (Housekeeping gene) as a control. All the experiment was performed in triplicates to govern the mean fold change expression values. The used primers in the experiment are listed in Table 1.

**Table 1:** Primers used for the RTPCR analysis of mRNA expression in zebrafish embryos exposed to BS, μP and BSμP.

Genes	Primers	Sequence (5′-3′)
actB1	Forward	GCGTGCACTGAAAACTCACA
	Reverse	GCAACTAGCTTGAAACTCGCC
sod1	Forward	ATTGAAATAGACGGTGCCGGT
	Reverse	CCTCATTGGTCGATTCCGCT
tp53	Forward	CTGTTTACACGCATTTGCCTTTT
	Reverse	TGCTCTGTAAACACGACCCGA

### 2.9 *In silico* analysis

To investigate the intricate molecular-level interaction mechanism a computational approach was employed [29]. The molecular interaction of BS and μP was scrutinized using the molecular docking approach. For the molecular docking procedure, both μP and BS were optimized by using the ATB server and AutoDock module of MGL Tools. The docking process was conducted using the AutoDock Vina (Version 1.5.7) with styrene (μP) as the ligand and BS as the receptor. The grid dimension was set to 18 × 14 × 12 with a spacing of 1 Å. The docked configuration results were analyzed by identifying the ideal binding position with the lowest binding energy. Post-docking analysis and visualization of the receptor-ligand interaction were performed with the help of Discovery Studio Visualizer and ChimeraX.

For mechanistic toxicological analysis of BS and μP, molecular interaction was done individually with zhe1, sod1, and p53. An interaction study was performed by using AutoDock 4.2.6/AutoDock Tools 1.5.6 with BS and μP as ligands and zhe1, sod1, and p53 served as receptor proteins. The 3D structure of BS and μP were generated from the ATB server. Furthermore, their geometry was optimized, and also energy was minimized using the ATB server. PMV was used for energy minimization in all three receptor proteins (zhe1, sod1, and p53). The parameters for BS were set in Autodock 4.2.6. The grid dimensions for zhe1, sod1, and p53 were set to 40 × 54 x 46, 62 × 62 x 106, and 42 × 40 x 40 with all the protein receptors having a spacing of 1 Å. The docking was performed for the ligand-receptor complex (BS-receptor proteins). Similarly, the parameters were applied in Autodock 4.2.6 for μP acting as the ligand in interaction with all three receptor proteins [30]. The grid dimensions were configured as 40 × 56 x 46 for zhe1, 65 × 68 x 110 for sod1, and 42 × 38 x 40 for p53. Each of the protein receptors had a spacing of 1 Å. The docking was performed for the ligand-receptor complex (μP-receptor proteins). Subsequently, post-docking analysis was performed by the identification of optimal binding sites, characterized by the lowest binding energy and 0 rmsd value. Further analysis was done with the help of conformational clustering and visualized using Chimera and Discovery Studio Visualizer [31].

### 2.10 Statistical analyses

GraphPad Prism v9 (San Diego, California) was used for all types of statistical analyses. Respective confidential intervals were determined by the non-linear fit of the sigmoidal dose response curve. Data were analyzed by one way ANOVA followed by Tukey/*t*-test with significance set at P < 0.05. Correlation analysis was performed between ROS and apoptosis data by computing the non-parametric Spearman correlation.

## 3 Results and discussion

### 3.1 Synthesis and characterisation of biosurfactant-microplastic (BSμP) hybrid

**Fig. 1:**
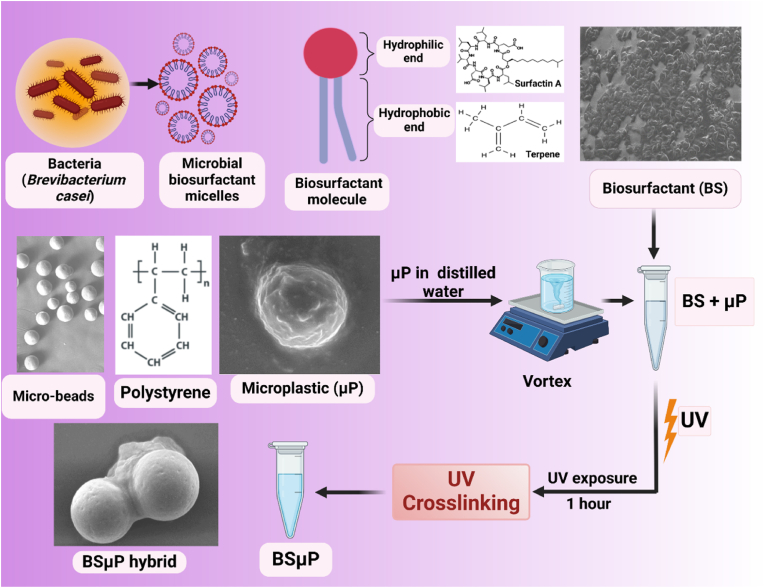
Schematic diagram displaying the green synthesis of Biosurfactant-polystyrene (BSμP) hybrid.

**Fig. 2:**
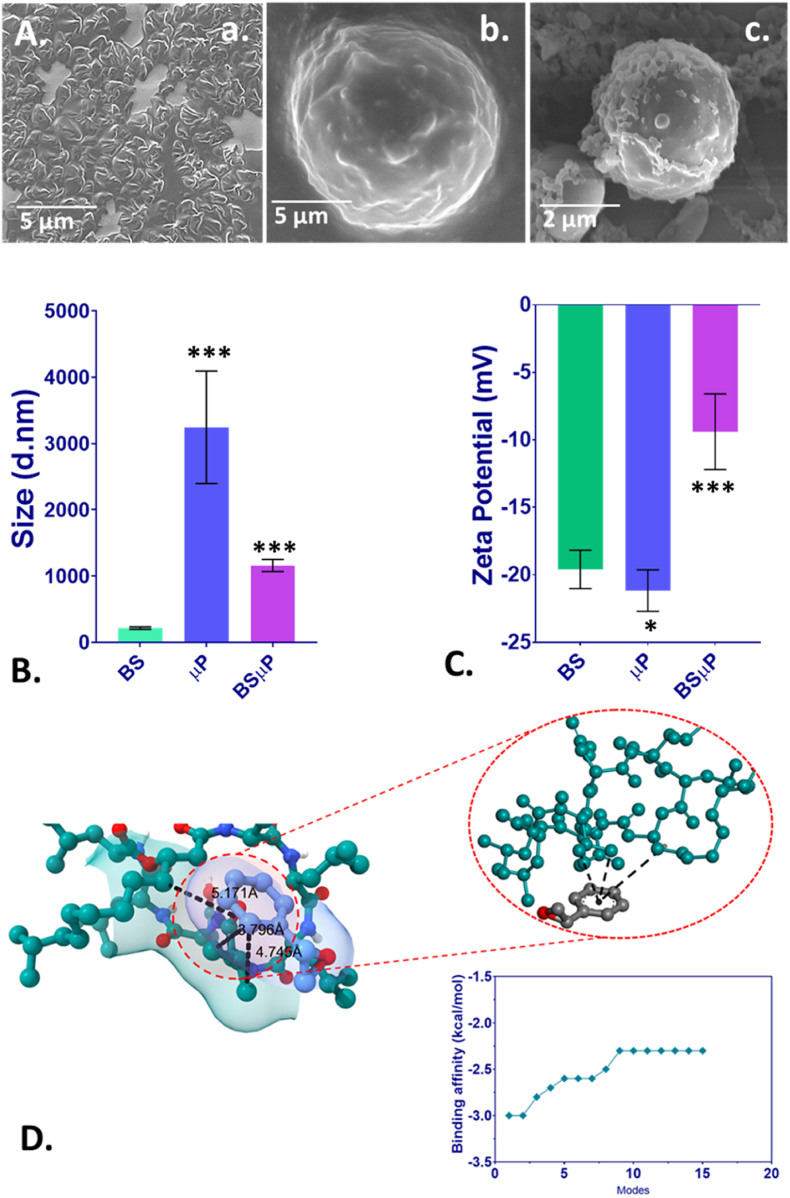
Physicochemical characterization of the BS, μP and BSμP; (A) Scanning electron microscopy images of (a) Biosurfactant (BS) (b) Polystyrene microplastic (μP) (c) BSμP; (B) hydrodynamic diameter of BS, μP, and BSμP determined by dynamic light scattering; (C) Zeta potential of BS, μP and BSμP determined by dynamic light scattering; the values are presented as Mean ± SD. ∗P > 0.5, ∗∗P > 0.01, and ∗∗∗P > 0.001 denote the compared significance compared to the BS as obtained from post hoc analysis after one-way ANOVA. ; (D) Molecular interaction of BS and μP in BSμP as determined by computational molecular docking.

The study was intended to understand the effect of biosurfactant exposure to microplastic and the consequent impact on the biotoxicity of microplastics. To comprehend, microplastic-biosurfactant hybrid was prepared at lab scale mimicking the natural process of UV exposure to the microplastic with biosurfactant. As shown in Fig. 1, well-characterized and purified microbial biosurfactant (BS) was exposed to UV light within an aqueous suspension containing microplastic beads (μP). UV light was supposed to act as catalyst in the formation of biosurfactant-microplastic hybrid (BSμP). The suspension was made using variable ratio of BS and μP to get the best optimized BSμP hybrid. Further, the prepared BSμP hybrid suspension was characterised for their physicochemical properties using standard physiochemical techniques. As shown in Fig. S1, the UV–Vis absorption spectra of BS, μP and the BSμP hybrid was found to be distinguished properly with an increasing variation of absorption peak. The variation in peak can be attributed to the formation of the BSμP hybrid. The prepared BSμP was further visualized through electron microscopy as shown in Fig. 2A and S2. The figures showed the formation of BSμP hybrid with an interpretation of the breaking of the microplastic bead in the presence of a higher volume of the biosurfactant. It was seen that the μP and BS were forming a hybrid with an increase in mixing ratio from 1:2 to 1:5. At the mixing ratio of 1:5 the microplastic beads were seen to be broken to form further tiny nanoplastics. The result can be attributed to the molecular action of biosurfactant to the microplastic beads [32], however, a detailed investigation is further needed for the conclusion of the degradation action of BS on μP.

This study focused on the formation of BSμP hybrid and its comparative impact on the biotoxicity of μP. Hence, the physicochemical properties like size and stability of the BSμP were determined to understand the physiological state of the hybrid. As shown in Fig. 2B and S3, the hydrodynamic size of the BSμP was found to be greater than BS clusters and was smaller than the μP. The reduced size of the BSμP can be reasoned to the molecular action of BS to μP in the aqueous medium [32]. It was intriguing that the stability of the BSμP would have been affected as the relative content of μP is decreased due to the incorporation of BS. The stability of the hybrid was further measured through determination of zeta potential to understand their persistence. As shown in Fig. 2C and S4, the zeta potential of the BSμP hybrid was found to be −10.3 ± 1.5 mV, while BS and μP possessed zeta potential of −19.6 ± 1.6 mV and −21.1 ± 1.4 mV. Moreover, the zeta potential of the BSμP was found to be dependent on the volume ratio of BS and μP used during the preparation of the BSμP. The result indicated the stability of BS, μP, and BSμP in the medium. The intriguing phenomenon of the formation of BSμP was further investigated at molecular level to understand the intrinsic interaction of molecules through computational analysis. As shown in Fig. 2D, the docking analysis showed intrinsic interaction of BS with polystyrene (the base component of μP) through π-alkyl and π-sigma bond with a bond length of 4.78 and 5.15 in case and π-alkyl interaction and 3.61 in case of π-sigma interaction (Table 2). These interactions can be reasoned to the formation of BSμP. Thus, the molecular and physicochemical analysis showed the formation of BSμP from BS and μP with a stable structural formation at the 1:2 (V/V) of BS: μP. The formed hybrid was further investigated for *in vivo* biotoxicity using the BSμP, formed at this specific mixture.

**Table 2:** Post docking analysis of interacting residue and their bonds.

BSμP Hybrid	Binding affinity (kcal/mol)	Type of bond formed (Distance in Å)	
	−3.0	Pi-Alkyl	4.78 and 5.15
		Pi-Sigma	3.61

### 3.2 *In vivo* biotoxicity of BS, μP and BSμP

**Fig. 3:**
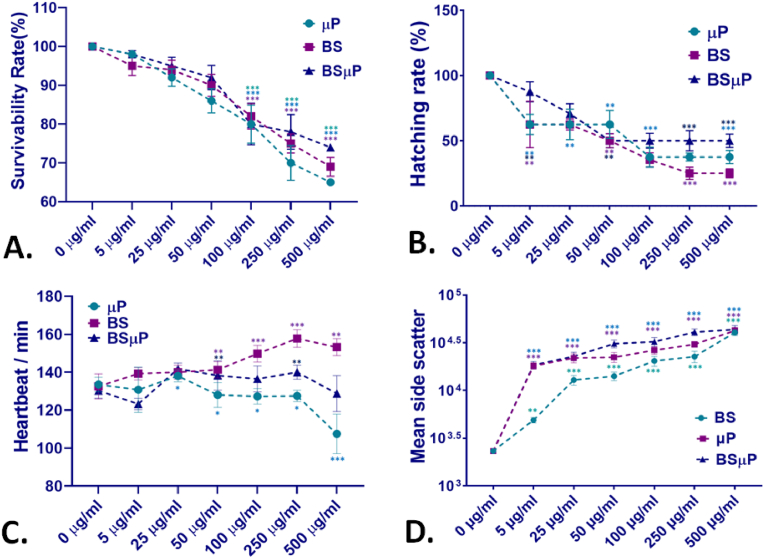
*In vivo* physiological toxicological effect of BS, μP and BSμP with embryonic zebrafish; (A) present the survivability rate of zebrafish embryos exposed to different concentrations of BS, μP and BSμP; (B) present hatching rate of zebrafish embryos exposed to different concentrations of BS, μP and BSμP; (C) present heartrate of zebrafish embryos exposed to different concentrations of BS, μP and BSμP. (D) Comparative mean side scatter of cellular suspension of 72 h exposed zebrafish embryos treated with different concentrations of BS, μP and BSμP. The values are presented as Mean ± SD of 20 embryos in triplicates. All the experimental analysis was done in triplicate and thrice independently. ∗P > 0.5, ∗∗P > 0.01, and ∗∗∗P > 0.001 denote the compared significant change at each exposed concentration as obtained from post hoc analysis after one-way ANOVA.

The *in vivo* biotoxicity of BS, μP, and BSμP was examined by studying their exposure impact on the cellular and physiological parameters of embryonic zebrafish. Firstly, the comparative survivability percentage of the embryos exposed to different concentrations of BS, μP, and BSμP was checked. The treatment concentration of BS, μP, and BSμP was chosen in accordance with the previously reported literature mentioning the confined toxicity of the BS and μP [33,34]. As shown in Fig. 3A, the survivability percentage of the embryos was found to be decreased with an increase in exposure concentration of all three materials BS, μP and BSμP. Comparatively, the survivability percentage was higher in the case of BSμP exposure than BS and μP at each exposed concentration. Specifically, it was also found that the survivability of embryos was higher in the case of BS than μP. The LC50 of BS, μP, and BSμP was calculated as 2.8 mg/mL, 2.3 mg/mL, and 3.4 mg/mL respectively. The data indicated to the expression of higher toxicity of μP and the reduction in toxicity of μP after conjugation with BS. Further, other physiological parameters were determined to understand the effect of BS, μP, and BSμP at the physiological level. As shown in Fig. 3B, concentration-dependent declination in hatching rate was found in embryos on exposure to BS, μP, and BSμP. However, the declination was less in the case of BSμP exposure compared to the BS and μP. Interestingly, BS was found to possess a severe effect on hatching rate in comparison of all three materials. The result can be attributed to the differential action of BS, μP, and BSμP on the hatching phenomenon through interaction with the hatching enzymes present in the chorion due to differential accumulation and attachment of BS, μP, and BSμP with the chorion surface [35,36]. Further, the heartbeat rate of the embryos exposed to BS, μP, and BSμP was examined. As shown in Fig. 3C, a concentration-dependent change in heartbeat rate was found in all three types of exposed embryos to BS, μP, and BSμP. Interestingly, acute changes were found at higher concentration exposure of μP and BS with a decreasing and increasing pattern respectively. However, there was no significant change in the case of BSμP exposure. The variation can be reasoned to the variable intensity of internalisation and accumulation of the BS, μP and BSμP because of their structural variation [37,38]. To crisscross the fact, uptake and accumulation analysis was performed through analysis of side scatter by flow cytometry in embryos cells exposed to different concentrations of BS, μP and BSμP. Fig. 3D displays the comparative mean side scatter intensity of BS, μP and BSμP in exposed embryos cells. The mean side scatter was found to be increased with increase in exposure concentration of BS, μP and BSμP. However, in case of BS and μP exposure, the mean side scatter was increased gradually with increased concentration while it was increasing initially and found to be consistent thereafter (Fig. S5). The change in pattern of the mean side scatter can be argued to the variability in attachment and internalisation [35].

**Fig. 4:**
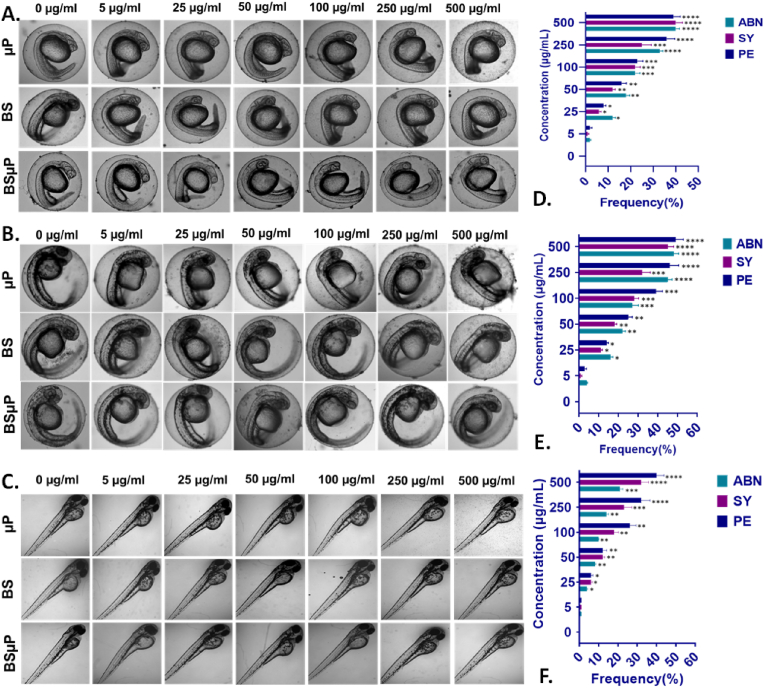
*In vivo* toxicological effect of BS, μP and BSμP with embryonic zebrafish; morphological abnormalities in zebrafish embryos exposed to different concentrations of BS, μP and BSμP exposed for (A) 24 h (B) 48 h (C) 72 h. The abnormalities frequency in zebrafish embryos exposed to different concentration of (D) BS, (E) μP and (F) BSμP; ABN: abnormal notochord, PE: pericardial edema, SY: swollen yolk. The values show Mean ± SD of 20 embryos in triplicates. All the experimental analysis was done in triplicate and thrice independently. ∗P > 0.5, ∗∗P > 0.01, and ∗∗∗P > 0.001 denote the compared significant change at each exposed concentration as obtained from post hoc analysis after one-way ANOVA.

The changes in physiological parameters in exposed embryos indicated to the phenomenon of disrupting the developmental process in the embryos. Hence, morphological change visualization was done in embryos exposed to Bs, μP, and BSμP. As shown in Fig. 4, morphological abnormalities like pericardial edema, abnormal notochord formation, and swollen yolk were found after 24h, 48h, and 72h of exposure to BS, μP, and BSμP with a concentration-dependent increasing frequency. Interestingly, the abnormalities frequencies were highest in case of μP exposure and least in case of BSμP exposure after 72 h of treatment. The data indicated the reduction of biotoxicity of μP after conjugation with BS and pointed to the variable attachment, accumulation, and internalisation of BS, μP and BSμP as a probable reason for the phenomenon [35].

**Fig. 5:**
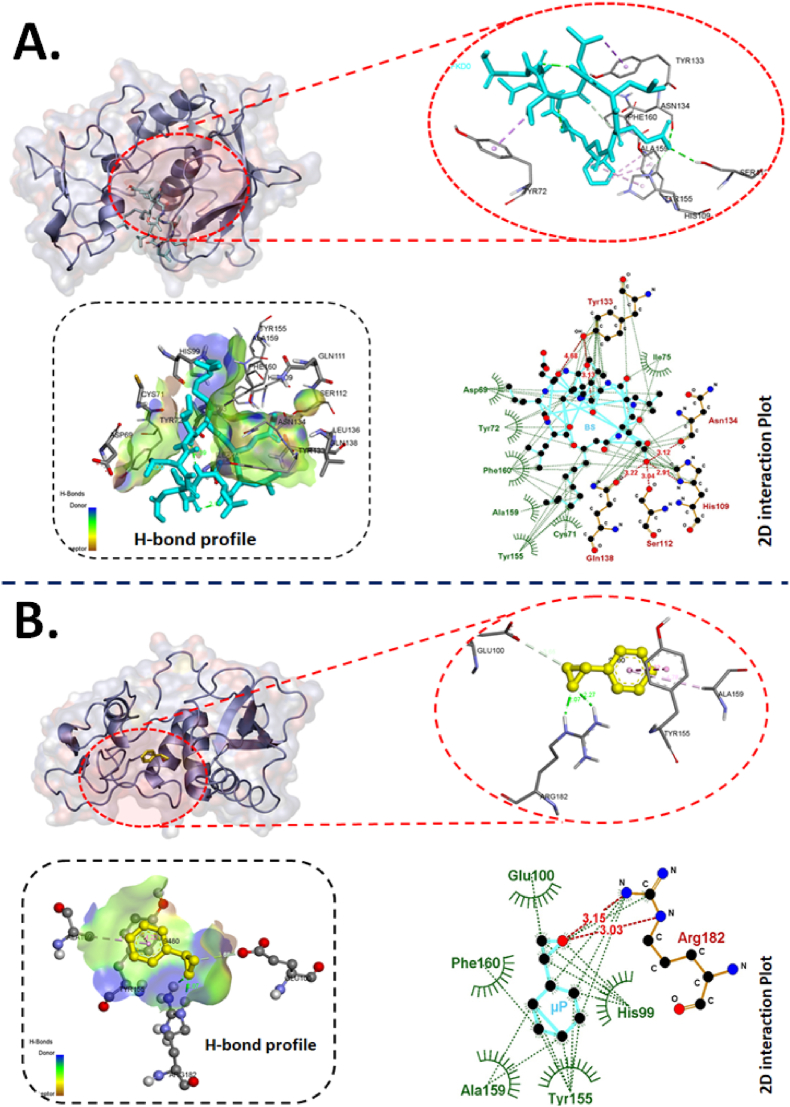
Computational analysis of molecular interaction of Zhe1a with BS and μP. (A) Conformational analysis of the interaction of Zhe1a enzyme with biosurfactant (BS) molecule. The diagram presents bond interaction analysis of BS with Zhe1a at best conformational site and LigPlot+ 2D presentation of interaction bond with amino acid residues of Zhe1a with BS. (B) Conformational analysis of the interaction of Zhe1a enzyme with styrene (μP). the diagram shows bond interaction analysis of μP with Zhe1a at best conformational site. LigPlot+ 2D presentation of interaction bond with amino acid residues of Zhe1a with μP.

The variability in attachment and accumulation of BS, μP and BSμP exhibited a change in hatching rate and other physiological parameters. Hence, it was speculated that the BS and μP would have been interacting with the molecular proteins and enzymes of chorion membrane of embryos to impact the hatching rate and the other consequences [30]. Fig. 5 shows the interaction of BS and μP with Zhe1 enzyme of chorion analyzed through computational molecular docking. The biosurfactant (BS) molecule was found to interact with Zhe1 enzyme intrinsically through different amino acids (Fig. 5A–S5) like tyrosine (TYR133), asparagine (ASN134), histidine (HIS 109), serine (SER112) and glutamine (GLN138) via H-bond with a bond length of 4.68 Å, 3.12 Å, 2.91 Å, 3.04 Å, 3.22 Å respectively. Additionally, hydrophobic interactions were also found with other amino acids of Zhe1. Similarly, polystyrene molecule of microplastic (μP) was interacting with amino acids glutamic acid (GLU 100), Arginine (ARG 182), tyrosine (TYR 155) and alanine (ALA 159) via H-bond with a bond length of 3.66 Å, 2.07 Å, 4.22 Å and 4.78 Å. These interactions can be attributed to influence the structural and functional integrity of the zhe1 enzyme [33]. Hence, it can be interpreted that the combinatorial effect of molecular interaction of BS and μP molecule with Zhe1 causes the hatching rate abnormalities in embryos exposed to BSμP.

### 3.3 *In vivo* cellular and molecular effect of BS, μP and BSμP

**Fig. 6:**
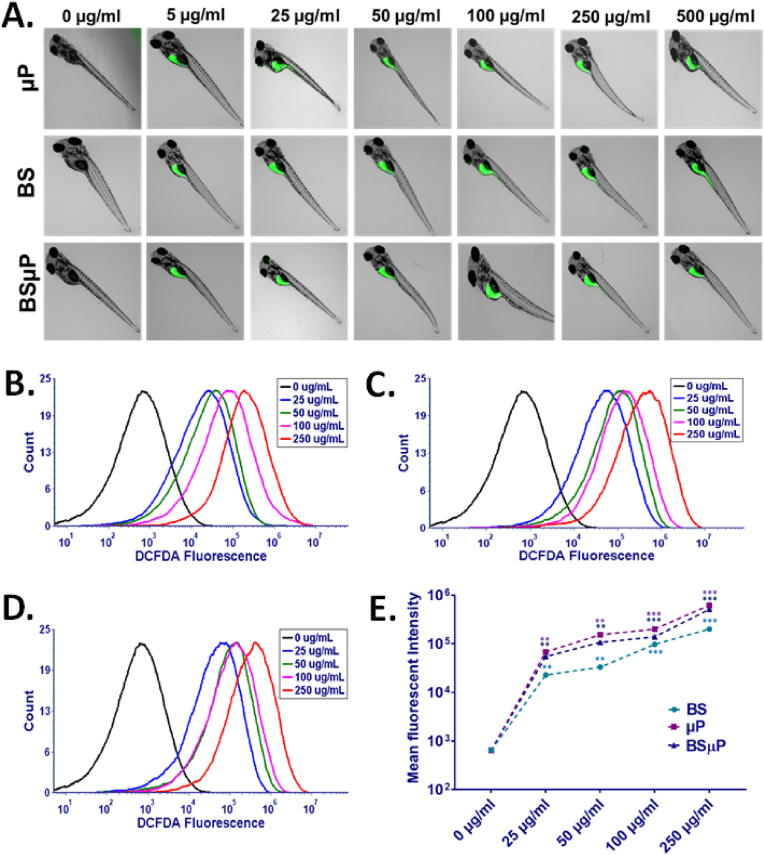
Cellular impact of BS, μP and BSμP; (A) Fluorescence image of zebrafish embryos exposed to BS, μP and BSμP for 72 h stained with DCFDA for oxidative stress evaluation. (B) Histogram presentation of DCFDA green fluorescence in zebrafish cells exposed for 72 h with different concentrations of BS (C) Histogram presentation of DCFDA green fluorescence in zebrafish cells exposed for 72 h with different concentrations of μP. (D) Histogram presentation of DCFDA green fluorescence in zebrafish cells exposed for 72 h with different concentrations of BSμP. (E) Comparative analysis of mean fluorescence intensity of DCFDA fluorescence in zebrafish cells exposed to BS, μP and BSμP for oxidative stress analysis. All the experimental analysis was done in triplicate and thrice independently. ∗P > 0.5, ∗∗P > 0.01, and ∗∗∗P > 0.001 denote the compared significant change at each exposed concentration as obtained from post hoc analysis after one-way ANOVA.

Experimental and computational interpretation of physiological disturbances in zebrafish embryos revealed concentration-based abnormalities in embryos exposed to BS, μP, and BSμP with a synergistic impact of BS and μP due to their differential accumulation and internalisation at the surface of the chorion. Moreover, the molecular interaction with the hatching enzyme Zhe1 showed a crucial role for the physiological changes. The accumulation and internalisation of xenobiotic compounds have been reported to induce cellular effects like abnormal oxidative stress, steatosis, and apoptosis for cellular biotoxicity [35,39,40,41]. Biosurfactants has been shown to exhibit oxidative stress in in vitro models like cell lines for their biocompatibility [33], similarly, many studies have explained the mediation of cellular toxicity of microplastic in *in vitro* and *in vivo* models through oxidative stress [36]. With reference to previous studies and the obtained experimental results, it was speculated that the changes in physiological parameters in zebrafish embryos due to BS, μP, and BSμP exposure would have been mediated through the cellular oxidative stress and consequently the induced apoptosis. The speculation was measured by experimental analysis of oxidative stress in BS, μP and BSμP exposed embryos through estimation of reactive oxygen species (ROS) by DCFDA stain green fluorescence. As shown in Fig. 6A, DCFDA green fluorescence intensity was found to be increased with an increase in exposure concentration of all three materials, BS, μP and BSμP. However, in comparison, the green fluorescence was least in the case of BSμP exposure than BS, μP alone. The fluorescent microscopy data was further validated quantitively through flow cytometry. As shown in Fig. 6B, C, and 6D, the mean fluorescent intensity of the DCFDA was seen to be increased with an increase in exposure concentration of BS, μP, and BSμP respectively. In comparison, it was observed that the mean fluorescent intensity in exposed embryos the fluorescent intensity was less in the case of BSμP exposure significantly (Fig. 6E). The flow cytometry analysis data supported the microscopy analysis data and confirmed the fact that, on exposure of BSμP to zebrafish embryos, the ROS induction becomes less than BS and μP exposure.

**Fig. 7:**
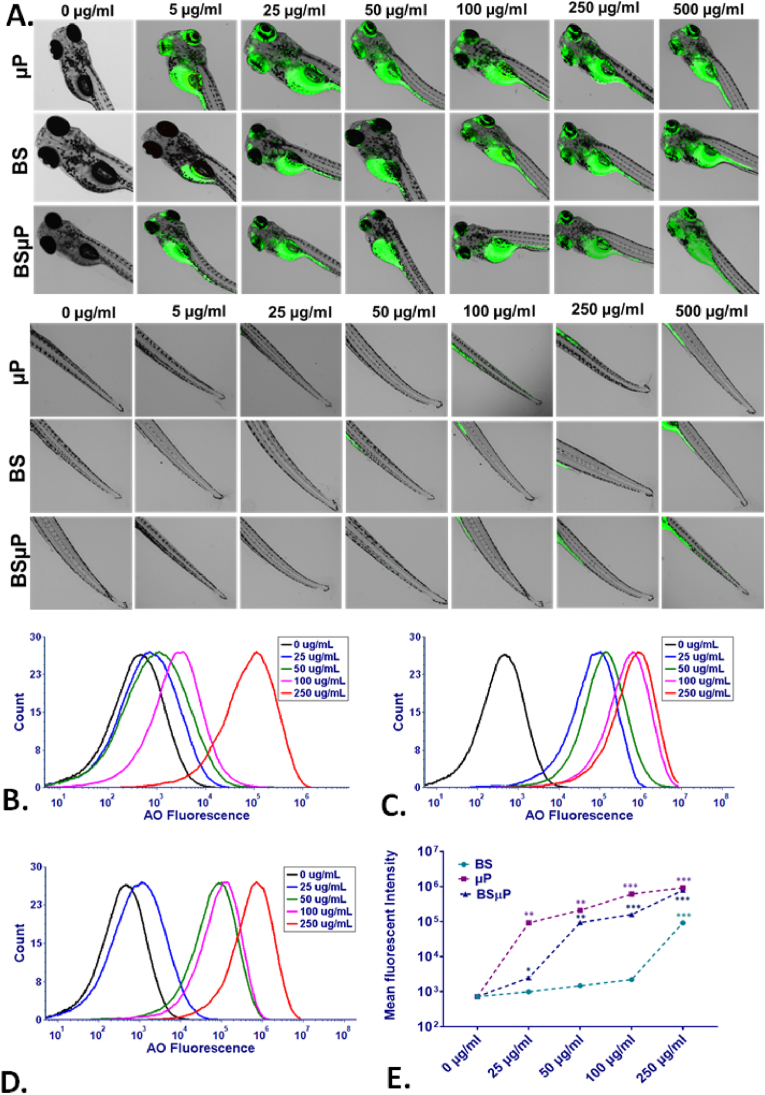
Cellular impact of BS, μP and BSμP; (A) Fluorescence image of zebrafish embryos exposed to BS, μP and BSμP for 72 h stained with Acridine orange for apoptosis evaluation. (B) Histogram presentation of Acridine orange green fluorescence in zebrafish cells exposed for 72 h with different concentrations of BS (C) Histogram presentation of Acridine orange green fluorescence in zebrafish cells exposed for 72 h with different concentrations of μP. (D) Histogram presentation of Acridine orange green fluorescence in zebrafish cells exposed for 72 h with different concentrations of BSμP. (E) Comparative analysis of mean fluorescence intensity of Acridine orange fluorescence in zebrafish cells exposed to BS, μP and BSμP for apoptosis analysis. All the experimental analysis was done in triplicate and thrice independently. ∗P > 0.5, ∗∗P > 0.01, and ∗∗∗P > 0.001 denote the compared significant change at each exposed concentration as obtained from post hoc analysis after one-way ANOVA.

The imbalance of ROS in cellular machinery has been reported as one of the key causes of cellular death phenomenon like apoptosis [42]. The differential imbalance ROS in zebrafish embryos exposed to BS, μP, and BSμP indicated the corelative apoptosis in embryos. The hypothesis was checked through the measurement of acridine orange's green fluorescence intensity in embryos exposed to BS, μP, and BSμP [43]. As shown in Fig. 7A, the green fluorescence was found to be increased with an increase in exposure concentration of BS, μP, and BSμP. Interestingly, it was least in the case of BSμP exposure than BS and μP. Moreover, it was the highest in embryos exposed to μP. The results were quantitively verified using flow cytometry [31]. Fig. 7B, C, and 7D show the mean fluorescent intensity of acridine orange in BS, μP and BSμP exposed embryos respectively. Comparatively, the mean fluorescent intensity was lowest in BSμP exposed embryos compared to BS and μP with the highest intensity on μP exposure (Fig. 7E). The results were in line with the previous works of literature explaining the induced apoptosis in zebrafish embryos exposed to emerging contaminants like microplastics and nanoplastics [36]. Moreover, the results were in correlation with the estimated ROS. The correlation analysis between the side scatter analysis, ROS and apoptosis data obtained by flow cytometry also confirmed the interrelation of these three phenomena (Figs. S6, S7, S8, S9).

**Fig. 8:**
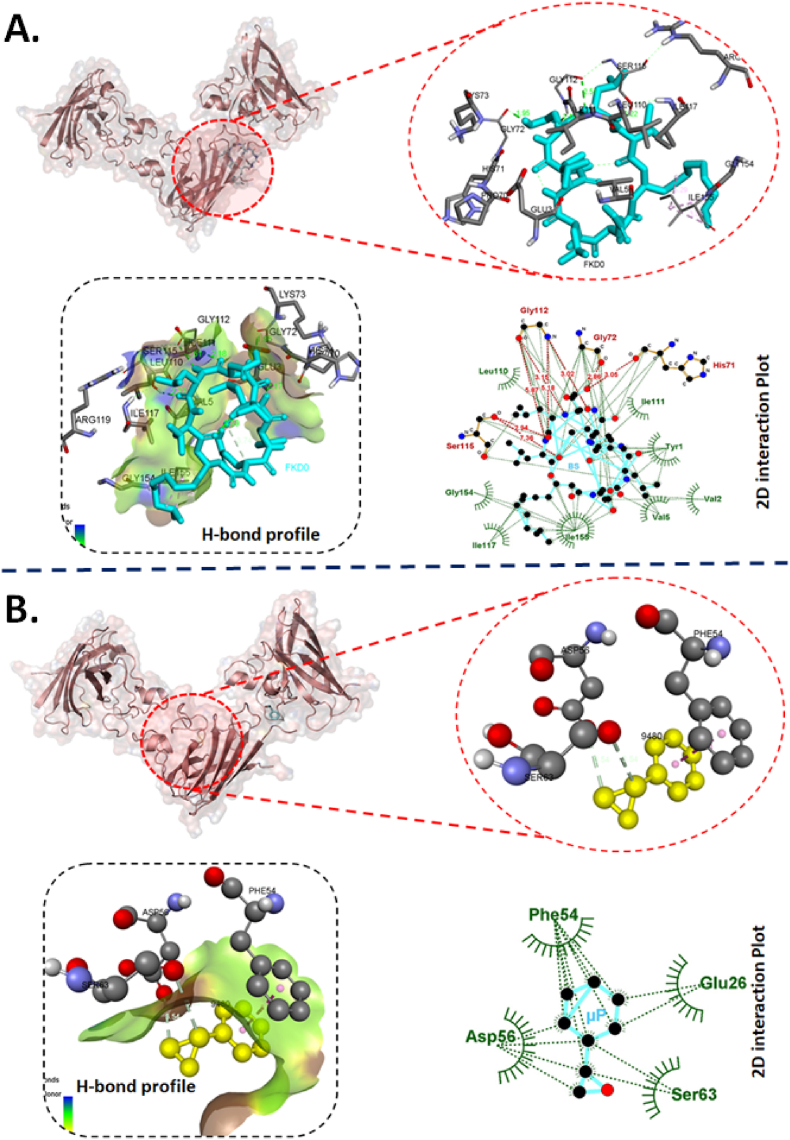
Computational analysis of molecular interaction of Sod1 with BS and μP. (A) Conformational analysis of the interaction of Sod1 enzyme with biosurfactant (BS) molecule. The diagram presents bond interaction analysis of BS with Sod1 at best conformational site and LigPlot+ 2D presentation of interaction bond with amino acid residues of Sod1 with BS. (B) Conformational analysis of the interaction of Sod1 enzyme with styrene (μP). the diagram shows bond interaction analysis of μP with Sod1 at best conformational site. LigPlot+ 2D presentation of interaction bond with amino acid residues of Sod1 with μP.

**Fig. 9:**
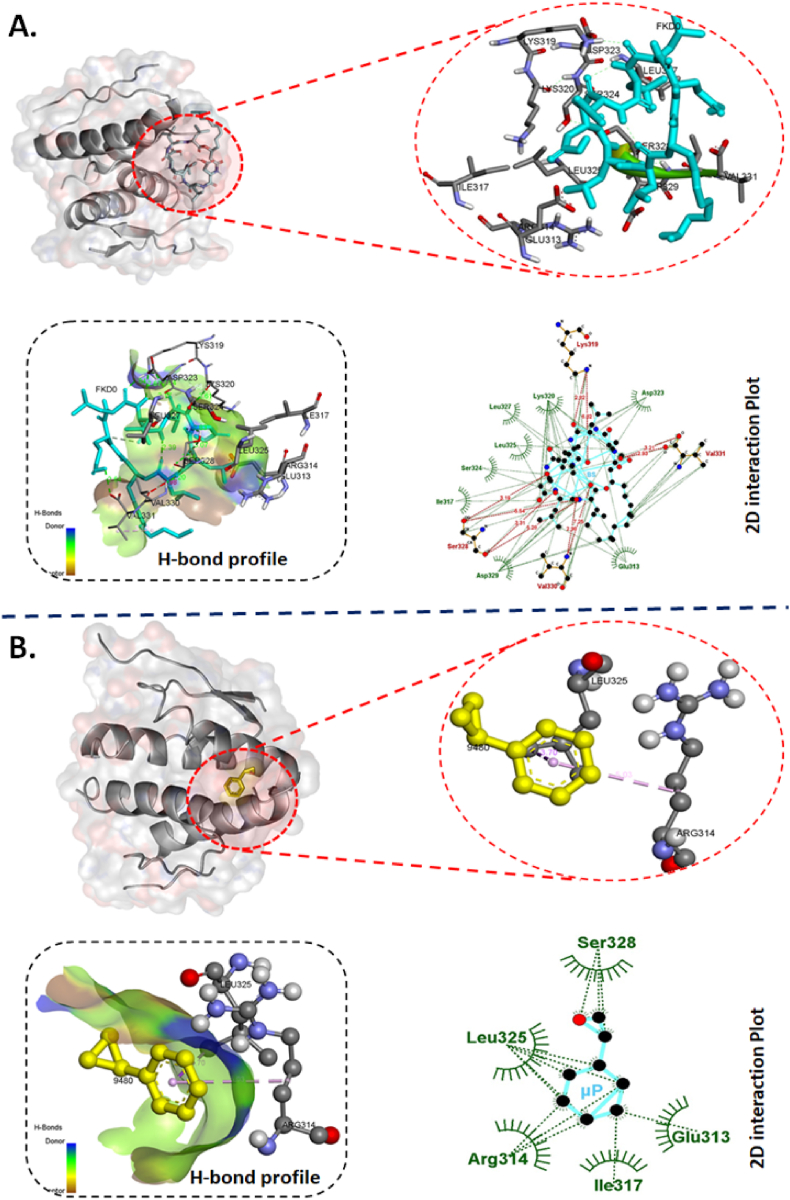
Computational analysis of molecular interaction of p53 with BS and μP. (A) Conformational analysis of the interaction of p53 enzyme with biosurfactant (BS) molecule. The diagram presents bond interaction analysis of BS with Sod1 at best conformational site and LigPlot+ 2D presentation of interaction bond with amino acid residues of p53 with BS. (B) Conformational analysis of the interaction of p53 enzyme with styrene (μP). the diagram shows bond interaction analysis of μP with p53 at best conformational site. LigPlot+ 2D presentation of interaction bond with amino acid residues of p53 with μP.

**Fig. 10:**
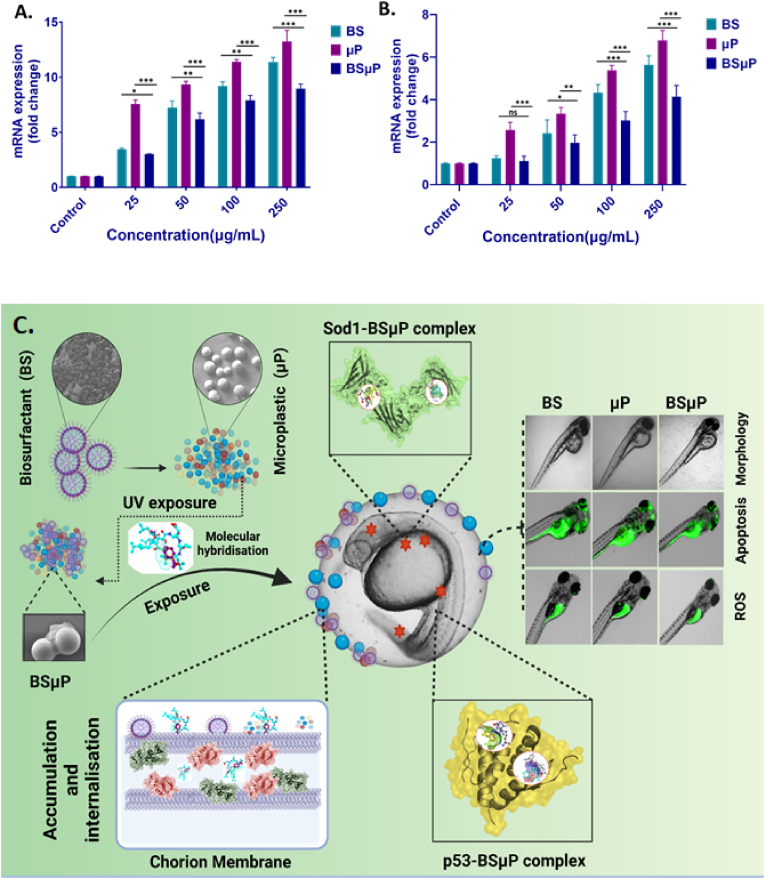
*In vivo* impact of BS, μP and BSμP on zebrafish embryos; (A) Fold change expression of mRNA expression of Sod1 in embryonic zebrafish cells exposed to BS, μP and BSμP as determined by RTPCR analysis. (B) Fold-change expression of mRNA expression of p53 in embryonic zebrafish cells exposed to BS, μP and BSμP as determined by RTPCR analysis. The values represent the mean ± SD of three independent experiments. ∗P > 0.5, ∗∗P > 0.01, and ∗∗∗P > 0.001 denote the compared significant change at each exposed concentration as obtained from post hoc analysis after one-way ANOVA. (C) Schematic presentation of the mechanistic biotoxicity of Biosurfactant-polystyrene hybrid (BSμP) with zebrafish.

The cellular analysis of ROS and apoptosis defined the differential toxicity of BS, μP and BSμP and entailed the information of reduction of biotoxicity of μP by hybridizing with BS. However, the molecular mechanism remained uncovered. Previous reports have defined the molecular effect of biotoxicity induced by ROS and apoptosis in embryonic zebrafish by different xenobiotics like nanomaterials [26] and pesticides [29,40] because of the abnormal protein expression and gene regulation like superoxide dismutase (Sod1) and p53. Moreover, the phenomenon has been defined to be regulated by the molecular interaction of the xenobiotic molecules with these proteins. It was speculated that the biosurfactant and microplastics were interacting with Sod1 and p53 at the molecular level to induce ROS and apoptosis. The hypothesis was checked experimentally and computationally in detail to uncover the fact. As shown in Fig. 8 and Table 3, molecular docking analysis showed the interaction of BS with Sod1 via amino acids like glycine (GLY72), serine (SER115) and histidine (HIS71) via strong H-bond (Fig. 8A) with binding affinity −5.8 kcal/mol. Similarly, μ was found to interact with Sod1 through amino acids phenylalanine (PHE54), Glutamine (GLU26), Asparagine (ASP56) and Serine (SER63) via hydrophobic interactions (Fig. 8B) with binding affinity of −5.5 kcal/mol. Fig. 9 and Table 3 showed the molecular interaction of p53 with BS and μP and revealed that BS was interacting with p53 via amino acids lysine (LYS319), serine (SER328), and valine (VAL330, 331) via H-bond with a binding affinity of −5.5 kcal/mol. While, μP interaction with p53 was with amino acids like leucine (LEU325), Arginine (Arg314), isoleucine (Ile317), glutamine (GLU313), and seine (SER328) through hydrophobic interactions. The molecular interaction can be accounted for the influential structural disturbances and imbalance in functional regularity of the Sod1 and p53 leading to cellular phenomenon of disbalance in oxidative stress and apoptosis [30,35]. To verify, experimental analysis was done to check the expression of these two enzymes. As shown in Fig. 10A, the fold change of mRNA expression was found to be increased in BS, μP and BSμP exposed embryos with increase in exposure concentration. Interestingly, the fold change expression was highest in case of μP exposure while lowest in case of BSμP exposure. Moreover, there was a significant fold change of expression on BSμP exposure compared to μP and BS in a decreasing manner. The result verified the hypothesis and indicated that the biotoxicity of μP get reduced at molecular level after being hybridised with BS. The overall study signified to the mechanistic influential reduction in *in vivo* biotoxicity of microplastic by hybrid formation with BS.

**Table 3:** Post docking analysis of interacting residue and their bonds.

Sl.No	Ligand	Protein	Binding affinity (kcal/mol)	Residues participating in hydrophobic interaction	Residues forming hydrogen-bond (Bond length in Å)	
1	BS	zhe1	−7.4	Asp69, Tyr72, Phe160, Ala159, Tyr 155, Cys71, Ile75	Tyr133	4.68 and 3.13
					Asn134	3.12
					His109	2.91
					Ser112	3.04
					Gln138	3.22
2	BS	p53	−5.5	Lys320, Leu327, Leu325, Ser324, Ile317, Asp329, Glu313, Asp323	Lys319	2.28 and 6.28
					Val330	2.98 and 7.25
					Ser328	3.19, 3.31, 5.28 and 5.54
					Val331	2.93 and 3.21
3	BS	sod1	−5.8	Leu110, Gly154, Ile117, Ile155, Val5, Val2, Tyr1, Ile111	His71	3.05
					Gly72	2.86
					Gly112	3.02, 3.15, 5.18 and 5.87
					Ser115	2.94 and 7.36
4	μP	zhe1	−4.6	Glu100, Phe160, Ala159, Tyr155, His99	Arg182	3.03 and 3.15
5	μP	p53	−4.4	Leu325, Arg314, Ile317, Glu313, Ser328		
6	μP	sod1	−4.3	Phe54, Asp56, Ser63, Glu26

### 3.4 Mechanism

With the help of computational and experimental approach, the study explained in detail about the mechanistic comparative biotoxicity of BS, μP and BSμP with embryonic zebrafish model. The experimental results showed comparative and discrepant abnormalities in physiological parameters like hatching rate, survivability rate and heartbeat rate along with cellular parameters like oxidative stress and apoptosis. The computational results elucidated the molecular interaction of BS and μP for the formation of BSμP hybrid and firm intrinsic interaction of BS and μP with metabolic proteins like zhe1a, sod1 and p53. Referred to the previous literature and this study results, the mechanistic discrepancy in *in vivo* biotoxicity of BS, μP and BSμP can be caricatured as an effect of induced ROS and apoptosis in exposed embryonic zebrafish cells due to combinatorial effect of their accumulation at the surface of chorion and internalisation inside the cells [29,30,35]. BS, μP and BSμP get accumulated at the surface of chorion leading to blockage of the chorion pores. The blockage creates a hypoxic condition inside the embryo thus unbalancing the cellular machinery of oxidative stress leading to higher ROS production. Moreover, the interaction of accumulated BS, μP and BSμP particles with zhe1a hatching enzyme also disrupt the chorion hardening mechanism leading to change in chorion pore size. This leads to the internalisation of bigger sized molecules of μP and BSμP. The internalised BS, μP and BSμP particles interact with metabolic proteins Sod1 and p53 leading to influential changes in their structural and functional integrity. The combinatorial effect of disruption in cellular machinery due to hypoxic conditions and enzymatic regulation leads to the induction of oxidative stress and results into apoptosis phenomenon. The discrepancy in the effect of BS, μP, and BSμP can be accounted to the differences in their physicochemical properties like shape, size, and stability. It can also be caricatured that the reduced effect of BSμP in comparison to BS and μP is because of the combined effect of BS and μP molecular interaction which would have been exhibiting the effects in a synergistic or antagonistic manner. However, the topic still remains open for further investigation. The detailed mechanistic elucidations can be visualized through the schematic diagram shown in Fig. 10B. The defined study was in line with the previous literature with a novel focus on the reduction of biotoxicity of microplastics through hybridisation with microbial biosurfactant.

## 4 Conclusion

In brief, the study entailed the discrepant *in vivo* biotoxicity of biosurfactant hybridised microplastic (BSμP) compared to biosurfactant (BS) and microplastic (μP) and defined the reduction of the toxicological effects of μP through hybridisation with BS. The hybridised BSμP was synthesized at lab scale through green process using UV light exposure to mimic the environmental process and characterized for their physicochemical properties. The physiochemical characterisation confirmed the formation of hybrid BSμP, which was found to be mediated by intrinsic atomic interaction between BS and μP. The hybrid was checked for their green biotoxicity in comparison to BS and μP using embryonic zebrafish model. The physiological parameter assessment including survivability rate, hatching percentage and heartbeat rate was found to be affected by the exposure of BS, μP, and BSμP, which minimum impact on BSμP exposure compared to BS and μP alone. The mechanistic molecular and cellular toxicity analysis unravelled the effect as a consequence of induced oxidative stress and apoptosis in embryos due to comparative accumulation and internalisation of BS, μP and BSμP in embryos with comparative gene expression due to molecular interaction with metabolic proteins like Zhe1, Sod1, and p53. The study deduced the comparative biotoxicity of BS, μP, and BSμP with a probable interpretation of reduction of biotoxicity of μP through hybridisation with BS.

The hybridization of microplastics (μP) with biosurfactants (BS) reduces the toxicological effects of microplastics. This suggests a viable strategy for mitigating the ecological damage caused by microplastics, which are pervasive in aquatic and terrestrial environments. Additionally, the laboratory synthesis of the hybridized BSμP via a green process using UV light mimics natural environmental conditions, offering a sustainable approach to addressing microplastic contamination. The lower impact of BSμP on survivability, hatching percentages, and heartbeat rates in embryonic zebrafish underscores its potential as a safer alternative for aquatic ecosystems. Hybridizing microplastics with biosurfactants could be applied in waste management strategies to reduce the environmental burden of μP pollution. Green processes offer a pathway for transforming harmful materials into less toxic forms. Moreover, the study provided invaluable inputs to the serious issue of microplastic pollution and a probable solution of using biosurfactants for the mitigation of the problem.

## CRediT authorship contribution statement

**Utsa Saha:** Writing – review & editing, Writing – original draft, Visualization, Validation, Methodology, Investigation, Formal analysis, Data curation, Conceptualization. **Aishee Ghosh:** Writing – review & editing, Writing – original draft, Visualization, Validation, Supervision, Resources, Methodology, Investigation, Funding acquisition, Formal analysis, Data curation, Conceptualization. **Adrija Sinha:** Writing – review & editing, Writing – original draft, Investigation, Formal analysis, Data curation, Conceptualization. **Aditya Nandi:** Writing – review & editing, Writing – original draft, Visualization, Formal analysis, Data curation, Conceptualization. **Sudakshya S. Lenka:** Writing – review & editing, Writing – original draft, Visualization, Software, Data curation, Conceptualization. **Abha Gupta:** Writing – review & editing, Writing – original draft, Visualization, Investigation, Formal analysis, Data curation, Conceptualization. **Shalini Kumari:** Data curation, Investigation, Methodology, Validation, Writing – review & editing. **Anu Yadav:** Writing – review & editing, Writing – original draft, Methodology, Investigation, Conceptualization. **Mrutyunjay Suar:** Writing – review & editing, Visualization, Validation, Supervision, Project administration, Investigation, Funding acquisition, Data curation, Conceptualization. **Nagendra Kumar Kaushik:** Data curation, Funding acquisition, Project administration, Supervision, Visualization, Writing – review & editing. **Vishakha Raina:** Writing – review & editing, Writing – original draft, Visualization, Supervision, Project administration, Investigation, Formal analysis, Data curation, Conceptualization. **Suresh K. Verma:** Writing – review & editing, Writing – original draft, Visualization, Validation, Supervision, Software, Resources, Project administration, Methodology, Investigation, Funding acquisition, Formal analysis, Data curation, Conceptualization.

## Declaration of Competing Interest

The authors declare that they have no known competing financial interests or personal relationships that could have appeared to influence the work reported in this paper.

## Data Availability

Data will be made available on request.
