# Supplementary material for: Intrinsic interaction inferred oxidative stress and apoptosis by Biosurfactant-microplastic hybrid reduces coordinated in vivo biotoxicity in zebrafish (Danio rerio)
Source: Mater Today Bio. 2025 Jan 7;31:101466. doi: 10.1016/j.mtbio.2025.101466 (PMC11966731; doi:10.1016/j.mtbio.2025.101466)
Supplement: Multimedia component 1 [file mmc1.docx]

**Supplementary Information**

**Intrinsic interaction inferred oxidative stress and apoptosis by Biosurfactant-microplastic hybrid reduces coordinated *in vivo* biotoxicity in zebrafish (*Danio rerio*).**

Utsa Saha^1^, Aishee Ghosh^2^*, Adrija Sinha^1^, Aditya Nandi^1^, Sudakshya S. Lenka^1^, Abha Gupta^1^, Shalini Kumari^3^, Anu Yadav^1^, Mrutyunjay Suar^1^, Nagendra Kumar Kaushik^4**^, Vishakha Raina^1**^, Suresh K. Verma^1^*

^1^School of Biotechnology, KIIT University, Bhubaneswar, India, 751024

^2^Department of Physics and Astronomy, Uppsala University, Box 516, Uppsala SE-751 20, Sweden

^3^Markham College of Commerce, Vinoba Bhave University, Hazaribagh, Jharkhand 825001, India

^4^Plasma Bioscience Research Center, Department of Electrical and Biological Physics, Kwangwoon University, 01897 Seoul, South Korea

*Correspondence: [sureshverma22@gmail.com](mailto:sureshverma22@gmail.com) , [aishee.ghosh@physics.uu.se](mailto:aishee.ghosh@physics.uu.se)

**Co-correspondence: [kaushik.nagendra@kw.ac.kr](mailto:kaushik.nagendra@kw.ac.kr) , [vraina@kiitbiotech.ac.in](mailto:vraina@kiitbiotech.ac.in).

**
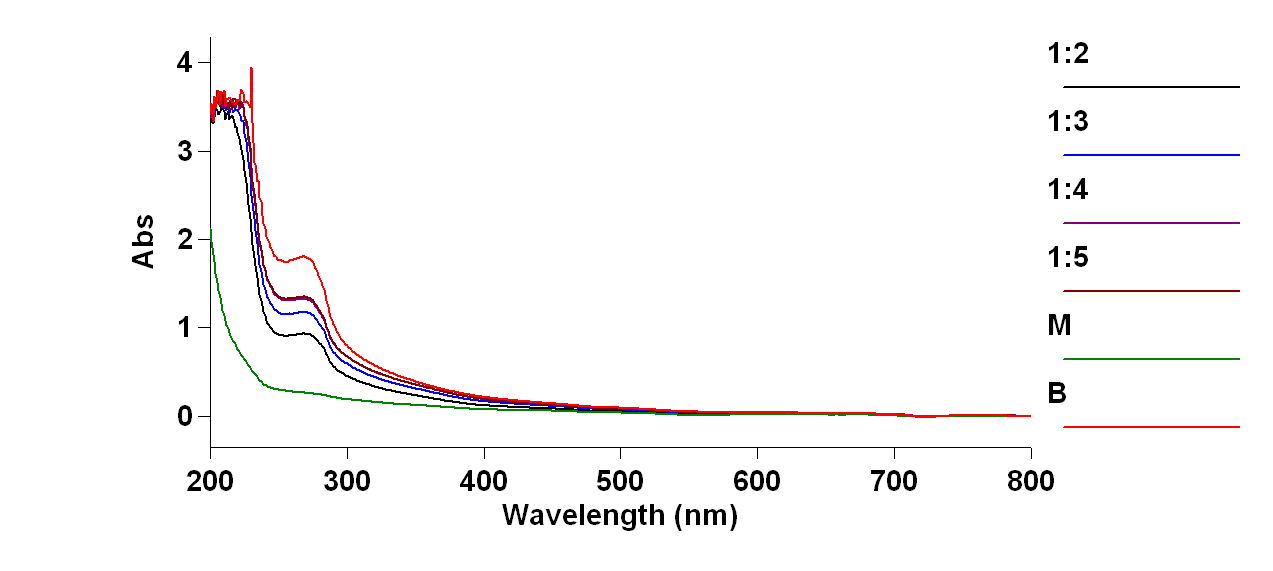
**

**Figure S1:** UV-Vis spectrum of BSµP hybrid analyzed by UV-Spectrophotometer. The ratio presents the (V:V) mixture of BS and µP used for the preparation of the hybrid.

**
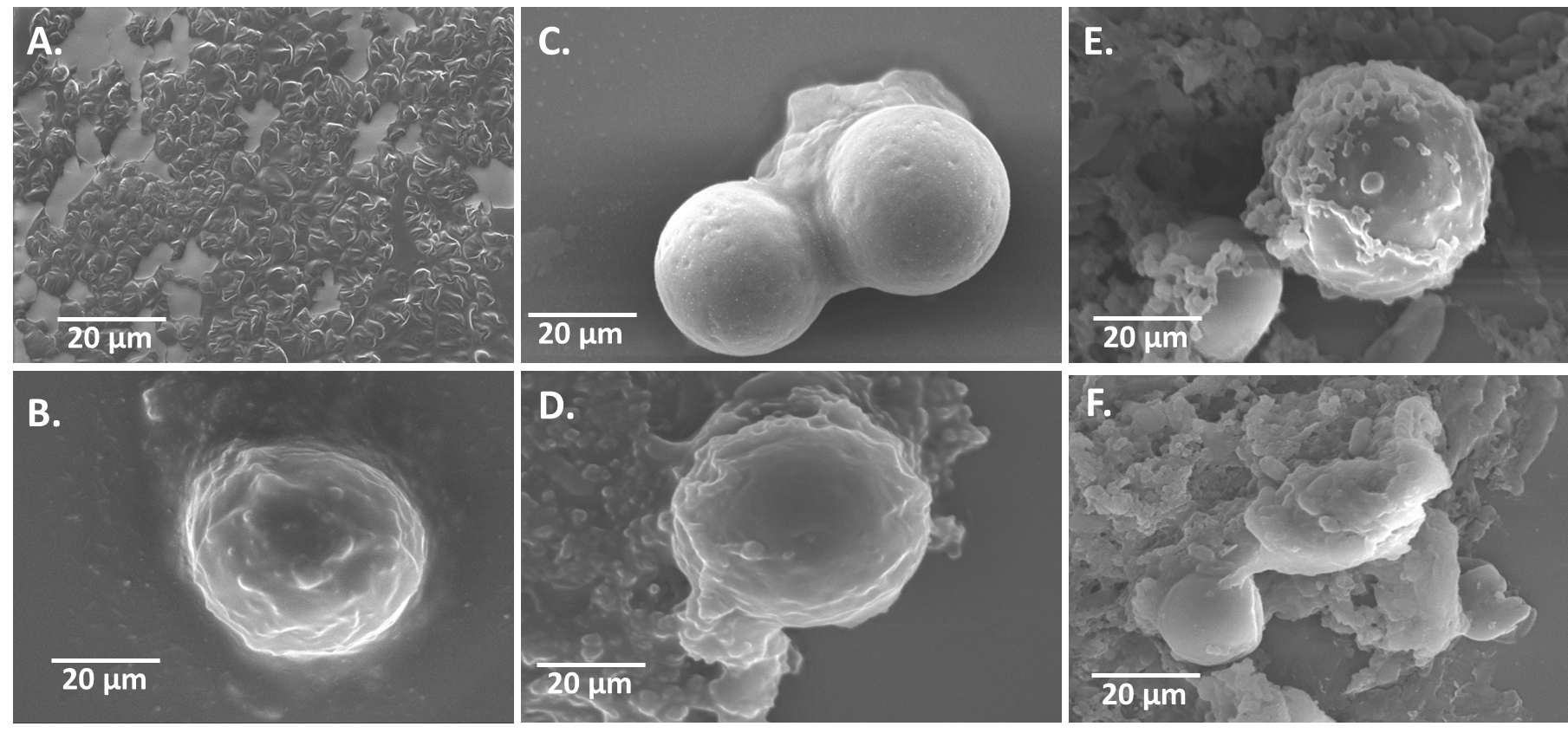
**

**Figure S2:** SEM image of native and hybridized synthesized materials;(A) Biosurfactant(BS) (B) Microplastic bead (µP) (C) 1:2 (BS:µP) (D) 1:3 (BS:µP) (E) 1:4 (BS:µP) (F) 1:5 (BS:µP).

**
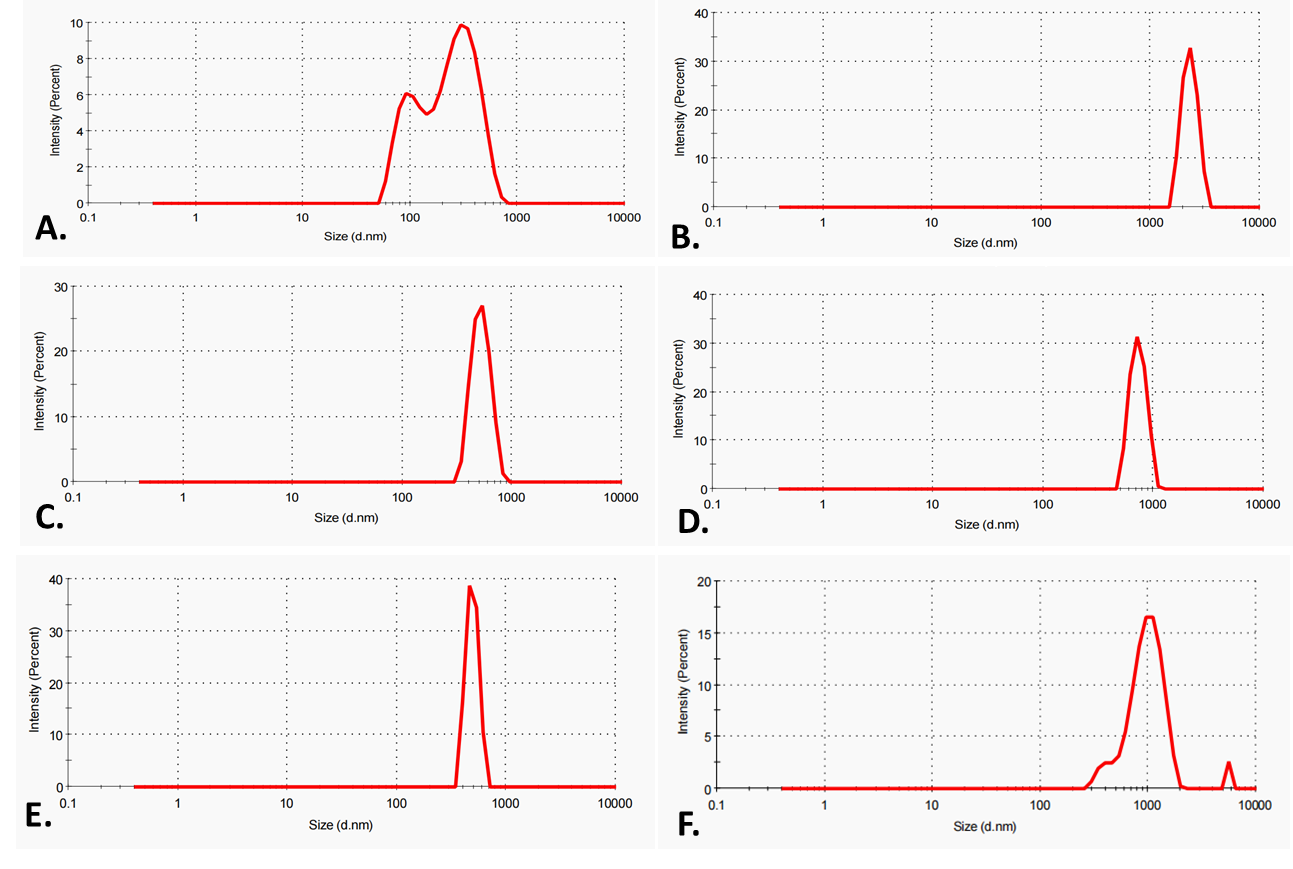
**

**Figure S3:** Hydrodynamic diameter of the materials determined by dynamic light scattering;(A) Biosurfactant(BS) (B) Microplastics bead (µP) (C) 1:2 (BS:µP) (D) 1:3 (BS:µP) (E) 1:4 (BS:µP) (F) 1:5 (BS:µP).

**
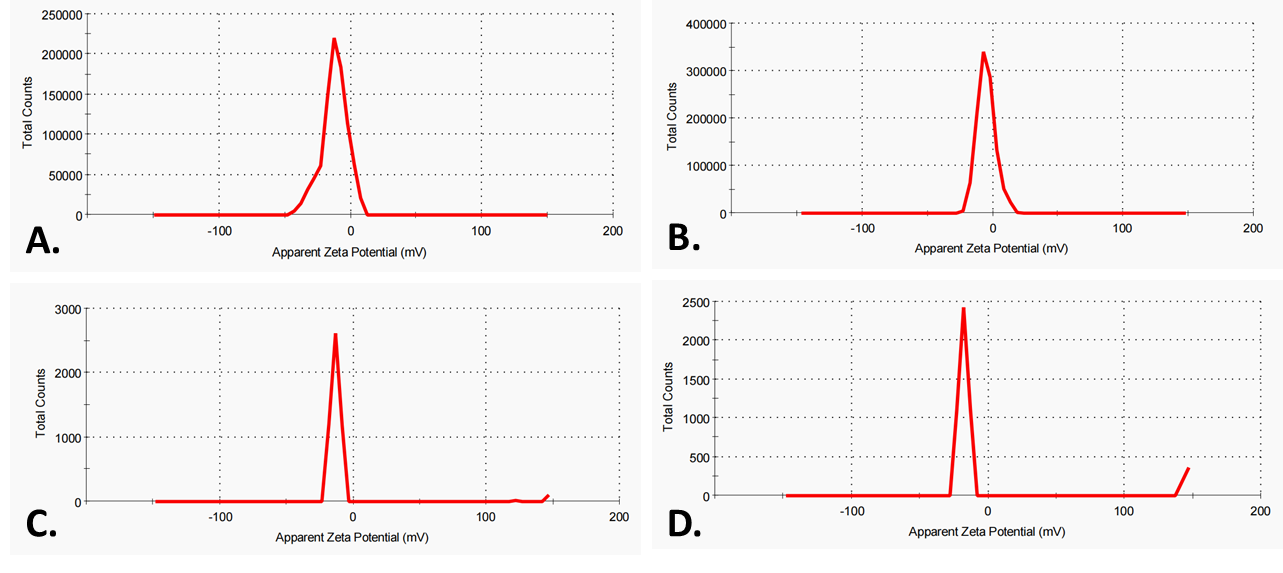
**

**Figure S4:** Zeta potential of the materials determined by dynamic light scattering;(A) Biosurfactant(BS) (B) Microplastics bead (µP) (C) 1:2 (BS: µP) (D) 1:3 (BS: µP) (E) 1:4 (BS: µP) (F) 1:5 (BS: µP).

**
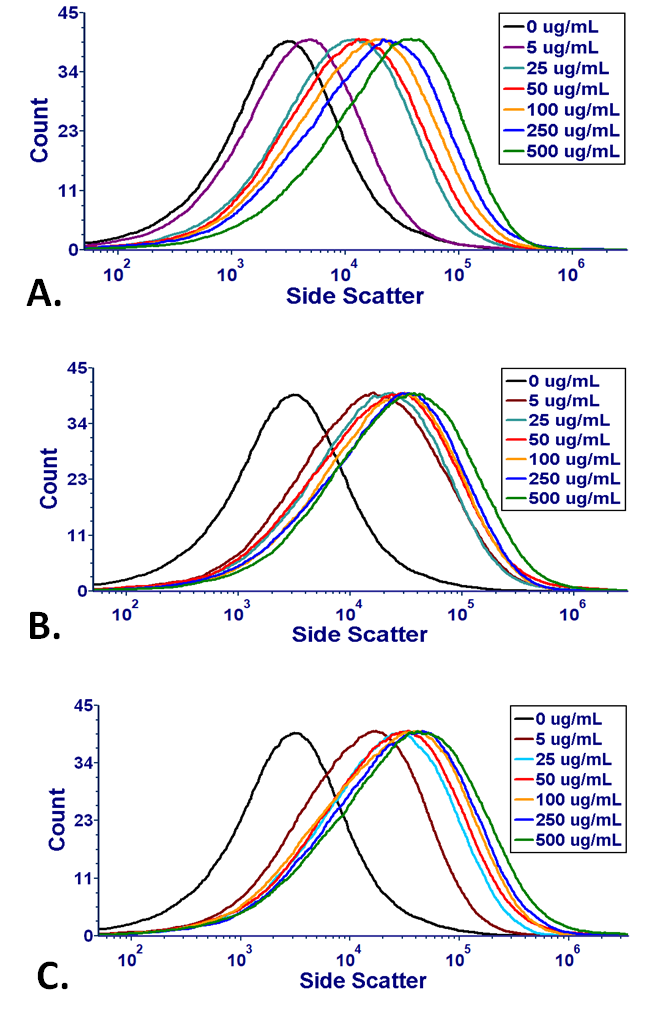
**

**Figure S5:** Cellular impact of BS, µP and BSµP; (A) Histogram presentation of Side Scatter of cellular suspension of 72 h exposed zebrafish embryos exposed to different concentrations of (A) BS (B) µP (C) BSµP.

**
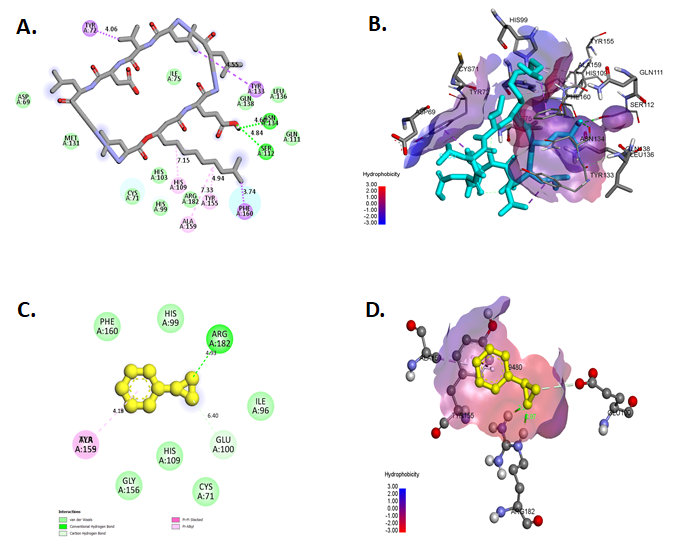
**

**Figure S6:** *In silico* interaction analysis of BS and µP with Zhe1a; (A) 2D plot of interaction of BS with Zhe1a (B) Hydrophobicity of the BS-Zhe1a interaction (C) 2D plot of interaction of µP with Zhe1a (E) Hydrophobicity of the µP -Zhe1a interaction.

**Figure S7:** Correlation analysis of uptake of BS and ROS as determined by side scatter analysis and DCFDA fluorescence analysis in BS exposed embryos.

**Figure S8:** Correlation analysis of uptake of µP and ROS as determined by side scatter analysis and DCFDA fluorescence analysis in µP exposed embryos.

**Figure S9:** Correlation analysis of uptake of BSµP and ROS as determined by side scatter analysis and DCFDA fluorescence analysis in BSµP exposed embryos.

**Figure S10:** Correlation analysis of uptake of BS and apoptosis as determined by side scatter analysis and Acridine orange fluorescence analysis in BS exposed embryos.

**Figure S11:** Correlation analysis of uptake of µP and apoptosis as determined by side scatter analysis and Acridine orange fluorescence analysis in µP exposed embryos.

**Figure S12:** Correlation analysis of uptake of BSµP and apoptosis as determined by side scatter analysis and Acridine orange fluorescence analysis in BSµP exposed embryos.

**
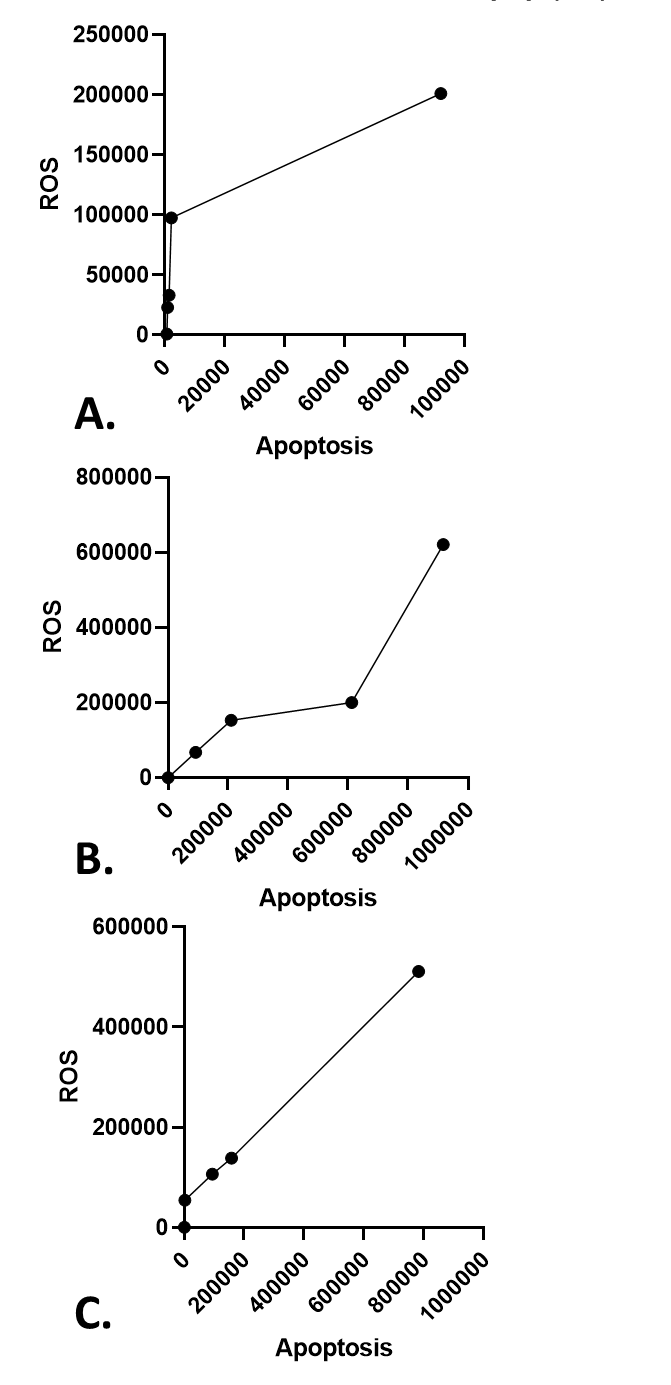
**

**Figure S13:** Correlation analysis of ROS and apoptosis as determined by flow cytometry analysis of DCFDA and Acridine orange fluorescence analysis in (A) BS (B) µP (C) BSµP exposed embryos.
